# Comparative analysis of codon usage patterns in chloroplast genomes of ten *Epimedium* species

**DOI:** 10.1186/s12863-023-01104-x

**Published:** 2023-01-09

**Authors:** Yingzhe Wang, Dacheng Jiang, Kun Guo, Lei Zhao, Fangfang Meng, Jinglei Xiao, Yuan Niu, Yunlong Sun

**Affiliations:** 1 College of Pharmacy, Jining Medical University, Rizhao, Shandong China; 2 School of Pharmaceutical Sciences, Changchun University of Chinese Medicine, Changchun, Jilin China; 3 Lanzhou Agro-Technical Research and Popularization Center, Lanzhou, Gansu China

**Keywords:** *Epimedium* species, Codon usage bias, Chloroplast genome, Mutation pressure, Natural selection

## Abstract

**Background:**

The Phenomenon of codon usage bias exists in the genomes of prokaryotes and eukaryotes. The codon usage pattern is affected by environmental factors, base mutation, gene flow and gene expression level, among which natural selection and mutation pressure are the main factors. The study of codon preference is an effective method to analyze the source of evolutionary driving forces in organisms. *Epimedium* species are perennial herbs with ornamental and medicinal value distributed worldwide. The chloroplast genome is self-replicating and maternally inherited which is usually used to study species evolution, gene expression and genetic transformation.

**Results:**

The results suggested that chloroplast genomes of *Epimedium* species preferred to use codons ending with A/U. 17 common high-frequency codons and 2–6 optimal codons were found in the chloroplast genomes of *Epimedium* species, respectively. According to the ENc-plot, PR2-plot and neutrality-plot, the formation of codon preference in *Epimedium* was affected by multiple factors, and natural selection was the dominant factor. By comparing the codon usage frequency with 4 common model organisms, it was found that *Arabidopsis thaliana, Populus trichocarpa,* and *Saccharomyces cerevisiae* were suitable exogenous expression receptors.

**Conclusion:**

The evolutionary driving force in the chloroplast genomes of 10 *Epimedium* species probably comes from mutation pressure. Our results provide an important theoretical basis for evolutionary analysis and transgenic research of chloroplast genes.

**Supplementary Information:**

The online version contains supplementary material available at 10.1186/s12863-023-01104-x.

## Background

The codons are composed of three adjacent nitrogen-containing bases on messenger RNA [1]. Codon is the link between nucleic acid and protein and plays an important role during the transmission of genetic information [2]. The genetic information carried by DNA is translated into amino acids in the form of triplet codons. Of the 64 codons, 61 are translated into 20 amino acids, and the other 3 are stop codons. Only methionine (Met) and tryptophan (Trp) are coded by one codon (AUG, UGG) respectively, and other amino acids are coded by 2–6 synonymous codons. The usage frequency of synonymous codons are different from prokaryotes to eukaryotes, which is due to the codons usage bias [3]. Codon usage preference is affected by environmental factors, base mutation, gene drift and gene expression level in the genomes of many organisms. In general, the main evolutionary driving force of codon use preference in microorganisms is mainly from mutation pressure, while in animals it is mainly from natural selection. But for plants, codon usage bias is affected by both natural selection and mutation pressure [4–7].

According to the Angiosperm Phylogenetic Group IV (APG IV) system, the genus *Epimedium* is the largest group under Berberidaceae [8]. More than 60 species of plants are widely distributed in Eastern Asia and Northwestern Africa, of which 50 species have been identified and mostly distributed in China [9]. The leaves of *Epimedium* have a medicinal history of more than 2000 years as herba “Yinyanghuo” in traditional Chinese medicine. The *Epimedium* plants bring great benefits to human health, containing antioxidant, anti-tumor and anti-osteoporosis. It has been proved by pharmacological research that the bioactive ingredients in *Epimedium* species are flavonol and its glycosides [10]. In plant taxonomy, *Epimedium* is one of the most taxonomically difficult resentatives of Berberidaceae. The number of *Epimedium* species increased rapidly within 40 years, but there is still a huge controversy in taxonomy due to the limited number of type specimens [11]. With the rapid development of sequencing and omics technology, chloroplast genome data of most species of *Epimedium* are released, which speed up the research progress of evolution and classification of species.

The chloroplasts are important organelles in plant cells that play a key role in photosynthesis. Compared with nuclear genome and mitochondrial genome, the chloroplast genome is special in structure and function, such as small size, highly conservative, simple structure and single parent inheritance. These characteristics have great advantages in genetic transformation. So it has attracted the attention of many scientists in recent years. Thanks to the advanced sequencing technology, more than 2000 plants chloroplast genomes have been published on NCBI, such as *Euphorbia* [12], *Jatropha* [13] and *Ricinus* [14]. There are three different types of chloroplast genes: photosynthesis genes, chloroplast expression genes and biosynthesis related genes. There have been many reports on the function of chloroplast genes in plants. For example, *sel1* mutation leads to etiolated plastid development defect [15], and *PTAC10* gene can affect chloroplast development and leaf color [16]. With the rapid development of transgenic technology, the method of chloroplast gene transformation has been developed and verified by many researchers. Seon Yeong Kwak et al. transferred chloroplast gene in mature *Eruca sativa*, *Nasturtium officinale*, *Nicotiana tabacum* and *Spinacia oleracea* plants using chitosan-complexed single-walled carbon nanotube carriers [17]. However, to construct a more stable transgenic system, it is necessary to study the codon usage pattern in plants.

In this study, we conducted a comparative analysis of the codon usage bias of chloroplast genomes in ten *Epimedium* species and discussed their causes of formation. Some parameters of codon preference had been calculated, such as the GC content of three positions (GC1, GC2, GC3), relative synonymous codon usage (RSCU), relative synonymous codon usage frequency (RFSC), and effective number of codons (ENc). All the chloroplast genomes of ten *Epimedium* species were analyzed, viz., *Epimedium koreanum* Nakai, *Epimedium acuminatum* Franch, *Epimedium hunanense* (Hand.-Mazz.) Hand.-Mazz, *Epimedium sagittatum* (Sieb. et Zucc.) Maxim, *Epimedium leptorrhizum* Stearn, *Epimedium pubescens* Maxim, *Epimedium myrianthum* Stearn, *Epimedium wushanense* Ying, *Epimedium brevicornu* Maxim. and *Epimedium coactum* H.R.Liang. This study will provide a reference for the research of genetic transformation and molecular evolution.

## Results

### Base composition analysis of chloroplast genomes in 10 *Epimedium* species

The number of CDS after filtered is 45, 57, 49, 52, 52, 47, 57, 46, 48, and 56 for *E. koreanum*, *E. acuminatum*, *E. hunanense*, *E. sagittatum*, *E. leptorrhizum*, *E. pubescens*, *E. myrianthum*, *E. wushanense*, *E. brevicornu,* and *E. coactum* respectively. The GC contents of chloroplast genomes of ten *Epimedium* species were calculated as shown in Table 1, and ranged from 38.82 to 39.08% with an average of 38.954%. The GC content of *E. koreanum* was the highest and the *E. acuminatum* was the lowest. Furthermore, the GC contents at the first (GC1), second (GC2), and third (GC3) position of codon were all less than 50%, it could be understood that the chloroplast genomes of ten *Epimedium* species prefer to use codons ending with A/U. Significantly, The highest value of GC1 was in *E. koreanum* and the lowest was in *E. acuminatum*, the highest value of GC2 was in *E. koreanum* and the lowest was in *E. pubescens*, the highest value of GC3 was in *E. wushanense* and the lowest was in *E. koreanum.* The GC content of three sites of 10 *Epimedium* species was different, but their distribution was in the trend of GC1 > GC2 > GC3.

**Table 1:** Base composition of codons in the chloroplast genome of ten *Epimedium* species. GC1, GC2 and GC3 represent the GC content at the first, second and third position; L_aa: the total number of amino acids

Species	GC%	GC1%	GC2%	GC3%	CDSs number (before filtering)	CDSs number (after filtering)	L_aa
*Epimedium koreanum*	39.08	47.15	38.23	31.84	80	45	20,018
*Epimedium acuminatum*	38.82	46.29	37.91	32.24	84	57	24,162
*Epimedium hunanense*	38.98	46.67	37.84	32.42	83	49	23,165
*Epimedium sagittatum*	38.95	46.60	37.90	32.34	83	52	23,442
*Epimedium leptorrhizum*	38.86	46.43	37.91	32.23	83	52	23,089
*Epimedium pubescens*	39.01	46.73	37.82	32.47	85	47	22,895
*Epimedium myrianthum*	38.91	46.44	37.99	32.29	83	57	23,917
*Epimedium wushanense*	39.01	46.71	37.83	32.48	83	46	22,797
*Epimedium brevicornu*	39.01	46.68	37.90	32.44	85	48	22,979
*Epimedium coactum*	38.91	46.47	37.97	32.29	83	56	23,858

### RCSU and RFSC analysis

According to Table S1, 26 common codons (RSCU > 1) were founded in 10 chloroplast genomes of *Epimedium* species, of which 25 codons ended with A/T (96.15%). There were 31 identical codons (RSCU< 1) among 10 chloroplast genomes of *Epimedium* species with 28 codons ending with G/C nucleotide (90.32%). The variation range of RSCU values in chloroplast genomes of each *Epimedium* plant was close, i.e. 0.39–1.81, 0.4–1.84, 0.39–1.82, 0.4–1.82, 0.38–1.84, 0.4–1.83, 0.4–1.81, 0.4–1.84, 0.4–1.84, 0.39–1.82 respectively (Table S1). The RSCU value of the codon AGA encoding Arginine was the highest, and the codon AGC encoding Serine was the lowest. A total of 17 common high-frequency codons were found in chloroplast genomes of ten *Epimedium* species, i.e. GCU, UGU, GAU, GAA, UUU, CAU, AAA, UUA, AUG, AAU, CCU, CAA, AGA, UCU, ACU, UGG, UAU (Table S1).

### Determination of putative optimal codons

The high and low expression datasets of genes were set up according to the ENc values of each CDS. Then the RSCU and ΔRSCU values were calculated by using CodonW 1.4.2 software as shown in Table S2. Furthermore, the optimal codons in ten *Epimedium* species were determined according to the ΔRSCU values, and the details were listed in Table 2. It is noteworthy that the CGU is the common optimal codon among ten *Epimedium* species, and the ACC is the common optimal codon among eight *Epimedium* species.

**Table 2:** The optimal codons in chloroplast genomes of ten *Epimedium* species

Species	Optimal codon numbers	Optimal codon
*Epimedium koreanum*	5	GUC, AGU, ACC, CGU, UGA
*Epimedium acuminatum*	5	UUC, CAC, CGU, UAA, UGA
*Epimedium hunanense*	4	AGU, ACC, CGU, UGA
*Epimedium sagittatum*	6	ACC, ACA, GCA, CAC, CGU, UGA
*Epimedium leptorrhizum*	5	ACA, GCA, GCG, CGU, UGA
*Epimedium pubescens*	4	AGU, ACC, CGU, UAA
*Epimedium myrianthum*	2	ACC, CGU
*Epimedium wushanense*	4	AGU, ACC, CGU, UGA
*Epimedium brevicornu*	4	AGU, ACC, CGU, UGA
*Epimedium coactum*	6	UUC, ACC, GCA, CAC, CGU, UGA

### Codon usage frequency

The results of comparative analysis of codon usage frequency in chloroplast genomes between ten *Epimedium* species and four commonly used exogenous expression hosts (*Escherichia coli*, *Saccharomyces cerevisiae*, *Arabidopsis thaliana*, and *Populus trichocarpa*) were shown in Table S3. The codon usage frequency of ten *Epimedium* plants was slightly different from that of *Arabidopsis thaliana, Populus trichocarpa,* and *Saccharomyces cerevisiae*, with 5–9, 6–8, and 7–9 different codons respectively. Nevertheless, The codon usage frequency of ten *Epimedium* plants was quite different from that of *Escherichia coli*, with 25–28 different codons. The codon usage frequency is closely related to the exogenous expression efficiency of chloroplast genes in *Epimedium* plants. Therefore, the *Arabidopsis thaliana, Populus trichocarpa,* and *Saccharomyces cerevisiae* were the best hosts for exogenous expression of chloroplast genes of *Epimedium* species. We also found that termination codons (UAA and UGA) are used differently.

### Source analysis of variation in codon usage patterns

#### ENc-plot analysis

**Fig. 1:**
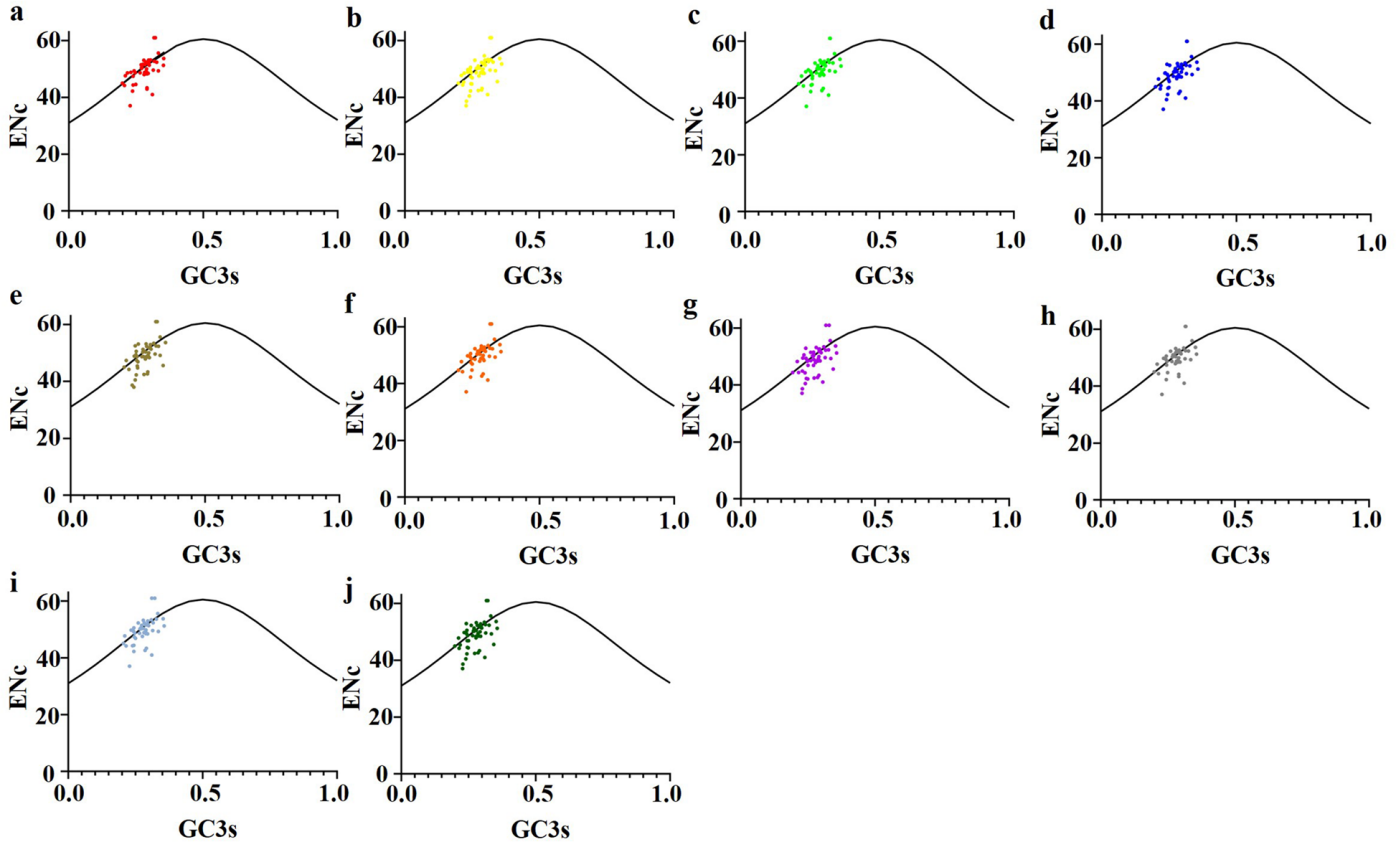
ENc-plot of chloroplast genomes of ten *Epimedium* species. (**a**) *Epimedium koreanum* (**b**) *Epimedium acuminatum* (**c**) *Epimedium hunanense* (**d**) *Epimedium sagittatum* (**e**) *Epimedium leptorrhizum* (**f**) *Epimedium pubescens* (**g**) *Epimedium myrianthum* (**h**) *Epimedium wushanense* (**i**) *Epimedium brevicornu* (**j**) *Epimedium coactum*

To analyze the codon usage variation in chloroplast genes, the ENc-GC3s plot analysis was performed as shown in Fig. 1. The distribution of CDSs of ten *Epimedium* species in the rectangular coordinate system was similar. A small number of CDSs were located above or near the expectation curve, which implied that the codon usage bias of chloroplast genomes was slightly affected by mutation pressure. However, most of the points are distributed below the desired curve, which indicated that natural selection played a major role in the formation of codon usage bias.

#### PR2-plot analysis

**Fig. 2:**
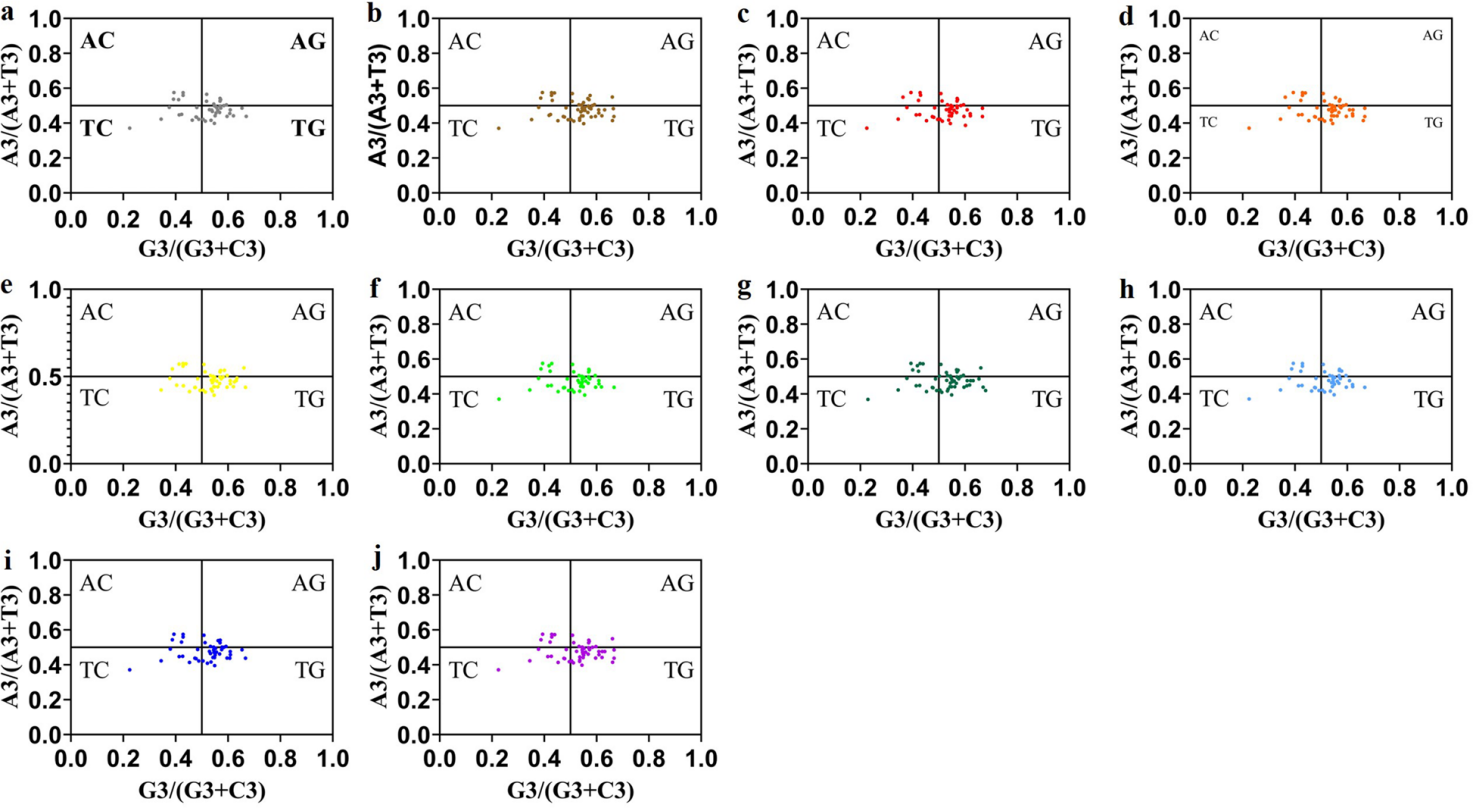
PR2-plot of chloroplast genomes of ten *Epimedium* species. (**a**) *Epimedium koreanum* (**b**) *Epimedium acuminatum* (**c**) *Epimedium hunanense* (**d**) *Epimedium sagittatum* (**e**) *Epimedium leptorrhizum* (**f**) *Epimedium pubescens* (**g**) *Epimedium myrianthum* (**h**) *Epimedium wushanense* (**i**) *Epimedium brevicornu* (**j**) *Epimedium coactum*

In this study, the points representing G3/(G3 + C3) and A3/(A3 + T3) values were distributed in scatter plots as shown in Fig. 2. The A/T(U)- bias was 0.477, 0.480, 0.475, 0.479, 0.481, 0.476, 0.479, 0.477, 0.476 and 0.479 for *E. koreanum, E. acuminatum, E. hunanense, E. sagittatum, E. leptorrhizum, E. pubescens, E. myrianthum, E. wushanense, E.brevicornu* and *E. coactum*, while the G/C-bias was 0.516, 0.523, 0.515, 0.515, 0.526, 0.510, 0.523, 0.510, 0.513 and 0.523, respectively. Meanwhile, the distribution of CDSs was not evenly around the center point (A = UT, G = C). Therefore, the formation of codon usage patterns are not only affected by mutation pressure, but also by natural selection.

#### Neutrality plot analysis

**Fig. 3:**
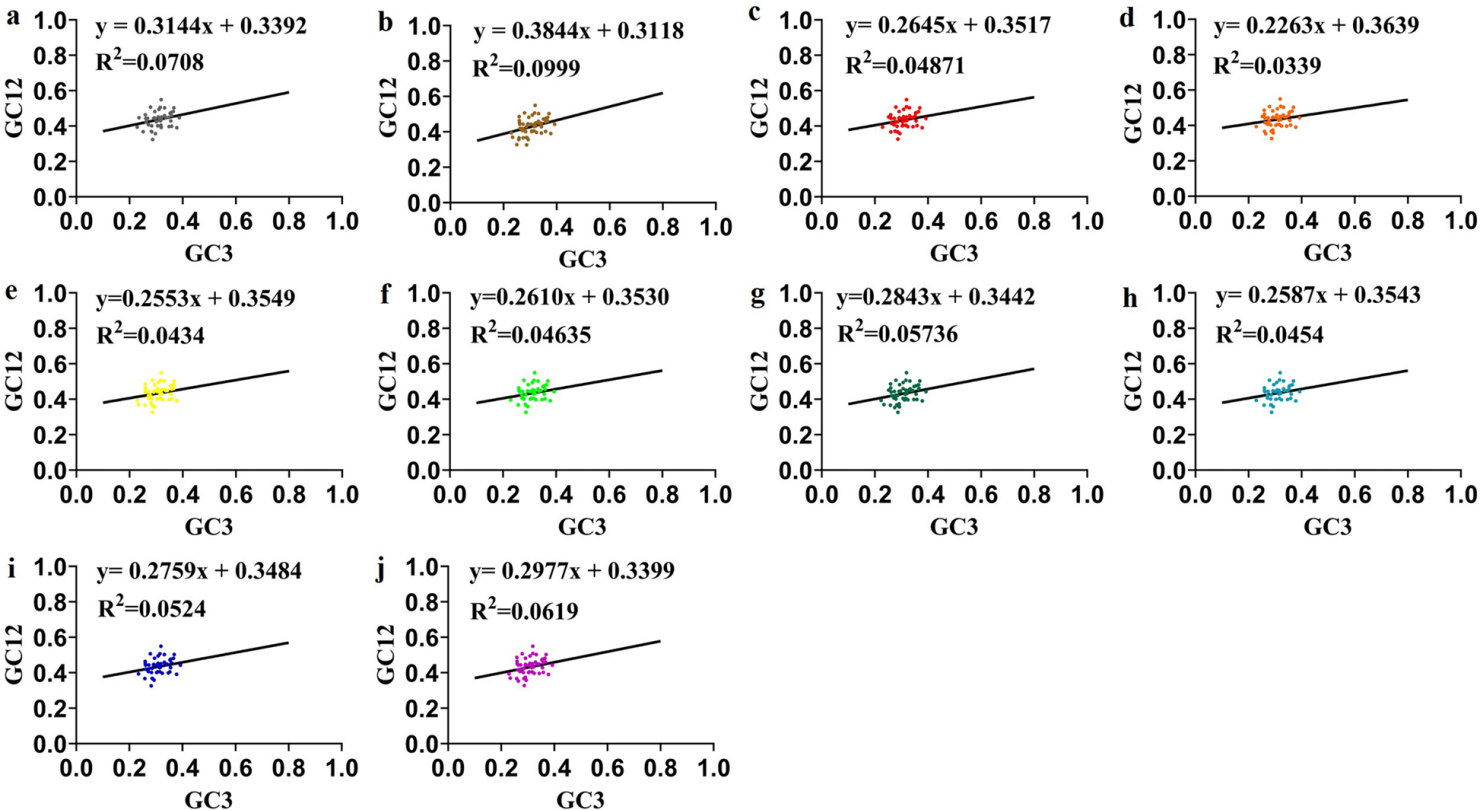
Neutrality plot of chloroplast genomes of ten *Epimedium* species. (**a**) *Epimedium koreanum* (**b**) *Epimedium acuminatum* (**c**) *Epimedium hunanense* (**d**) *Epimedium sagittatum* (**e**) *Epimedium leptorrhizum* (**f**) *Epimedium pubescens* (**g**) *Epimedium myrianthum* (**h**) *Epimedium wushanense* (**i**) *Epimedium brevicornu* (**j**) *Epimedium coactum*

In order to further investigate the degree and extent of mutation pressure and natural selection. The neutrality plots (regression of GC12 on GC3) were performed as seen in Fig. 3. According to the results, the correlation between GC12 and GC3 was analyzed and the effects of natural selection and mutation pressure on codon bias were discussed. The strong correlation between GC12 and GC3 values indicates that mutation pressure is the main factor in the formation of codon preference while the weak correlation between them indicates that natural selection is the main factor. The results of neutrality plots analysis of ten *Epimedium* species were very similar. The regression curve did not coincide with the diagonal in each graph, and the slope ranged from 0.2263 to 0.3844. Pearson correlation analysis showed that the correlation between GC1 and GC2 was significant, (r_1_ = 0.340, r_2_ = 0.339, r_3_ = 0.339, r_4_ = 0.355, r_5_ = 0.274, r_6_ = 0.390, r_7_ = 0.272, r_8_ = 0.408, r_9_ = 0.332, r_10_ = 0.292; *P*_1_ = 0.022, *P*_2_ = 0.010, *P*_3_ = 0.017, *P*_4_ = 0.010, *P*_5_ = 0.049, *P*_6_ = 0.007, *P*_7_ = 0.041, *P*_8_ = 0.005, *P*_9_ = 0.021, *P*_10_ = 0.029), and the correlation between GC1 and GC3 was not significant, (r_11_ = 0.318, r_12_ = 0.389, r_13_ = 0.280, r_14_ = 0.286, r_15_ = 0.271, r_16_ = 0.246, r_17_ = 0.312, r_18_ = 0.239, r_19_ = 0.291, r_20_ = 0.333; *P*_11_ = 0.033, *P*_12_ = 0.003, *P*_13_ = 0.052, *P*_14_ = 0.040, *P*_15_ = 0.052, *P*_16_ = 0.096, *P*_17_ = 0.108, *P*_18_ = 0.110, *P*_19_ = 0.045, *P*_20_ = 0.012). However, there was no correlation between GC2 and GC3, (r_21_ = 0.114, r_22_ = 0.113, r_23_ = 0.078, r_24_ = 0.012, r_25_ = 0.056, r_26_ = 0.113, r_27_ = 0.060, r_28_ = 0.117, r_29_ = 0.079, r_30_ = 0.053; *P*_21_ = 0.455, *P*_22_ = 0.401, *P*_23_ = 0.593, *P*_24_ = 0.934, *P*_25_ = 0.692, *P*_26_ = 0.450, *P*_27_ = 0.659, *P*_28_ = 0.439, *P*_29_ = 0.593, *P*_30_ = 0.699). This revealed that the codon usage bias of 10 *Epimedium* species was mainly affected by natural selection.

## Discussion

Codon usage bias is an important feature of genome evolution, which is of great significance for the study of molecular evolution and exogenous expression of genes [18]. The unequal use of synonymous codons varies in different organisms and genes. It has been found that codon usage bias is related to GC composition, tRNA abundance, gene expression level, and gene length [19]. Codon usage patterns and their possible causes have been studied in many species, for instance, in *Arabidopsis thaliana* [20], *Poncirus trifoliata* [21], *Gossypium hirsutum* [22], and many others.

The usage pattern of the codon is closely related to the GC content of the third base. Previous research has shown that the chloroplast genomes of dicotyledons generally prefer to use codons ending with A/U, but monocotyledons prefer to use codons ending with G/C [23]. Our study showed that the GC content and GC3 content of codons in ten *Epimedium* species were all less than 40%, indicating that codons preferred to end with A/U. This was consistent with previous studies. Chloroplast genomes in other plants, such as *Camellia amplexicaulis* [24], *Panicum incomtum* [25], *Oryza australiensis* [26], *Euphorbia esula* [27], etc., also tended to use codons ending with A/U. According to the RSCU analysis, it was found that most of the frequently used codons (RSCU > 1) were A/U-ending, whereas the less frequently used codons (RSCU< 1) were G/C-ending. This was consistent with the results of the base composition analysis.

The codon usage bias is mainly influenced by natural selection and mutation pressure [28]. However, The primary factors determining codon usage bias are different among many species. Neutrality plot analysis was used to analyze the correlation between the three codon sites. The variation trends of base composition at three sites of codon should be similar when mutational pressure is the main factor. On the contrary, when natural selection is the main factor, there is no correlation between the three codon sites [29]. In the current research, there was no significant correlation between GC12 and GC3 of chloroplast genomes in ten *Epimedium* species, demonstrating that natural selection played a dominant role in the formation of codon usage patterns [30].

Under the influence of mutation pressure, the base mutation probability at different positions of each codon is equal. The parity rule 2 analysis can reflect the difference in the use frequency of A, T, C and G at the third position of the codon [31]. According to the PR2-plot analysis of ten *Epimedium* species, the number of genes in the four quadrants was unevenly distributed. In the vertical direction, most genes were located below the midline. In the horizontal direction, the number of genes on the right was higher than that on the left. Therefore, G and T were used more frequently than C and A at the third position of codons. This indicated that natural selection was the main reason for the codon usage bias in chloroplast genomes of 10 *Epimedium* species [32].

The ENc-plot showed that the observed ENc values of a few genes were close to the expected values, indicating that codon bias of these genes was closely related to mutation pressure. The observed ENc values of most genes were smaller than expected, indicating that codon bias of these genes was closely related to natural selection [33].

Based on neutrality plot analysis, PR2-plot analysis and ENc-plot analysis, codon preference of chloroplast genomes of 10 *Epimedium* species was jointly affected by natural selection and mutation pressure, and natural selection played a leading role. Similar results were found in other plants such as *Miscanthus floridulus* [34], *Delphinium grandiflorum* [35] and *Hemiptelea davidii* [36] through chloroplast genome analysis. They all believe that natural selection was the main evolutionary driving force of chloroplast genome. Yue Gao et al. [37] analyzed the *Helianthus annuus* and found that the codon bias of chloroplast genome was mainly affected by mutation pressure. However, Guoling Li [38] and Supriyo Chakraborty [26] reported that codons of chloroplast genome of *Porphyra umbilicalis* and *Oryza* species were mainly influenced by natural selection. It can be seen from the above results that different genomes could be affected by various pressures, resulting in codon use preference.

The 2–6 optimal codons were found in the 10 species assessed here, and CGT is the consensus optimal codon among ten *Epimedium* plants. These results are meaningful for improving the expression efficiency of chloroplast genes in host cells. The heterologous expression host is also a considerable factor for genetic transformation and protein expression of chloroplast genes. After comparing the codon usage frequency of ten *Epimedium* species and four model organisms, we found that prokaryotic *E. coli* was not suitable for a heterologous expression host for *Epimedium* chloroplast genes. However, due to the small number of differential codons, the eukaryotes *A.thaliana, P. trichocarpa,* and *S. cerevisiae* were suggested as exogenous expression hosts for chloroplast genes of the ten *Epimedium species* [39].

## Conclusions

In the study, 509 CDSs were chosen to analyze the codon usage bias in the chloroplast genome of 10 *Epimedium* species by the CodonW1.4.2 program. According to base composition and RSCU analysis, ten *Epimedium* plants preferred to use codons ending with A/U. The possible reasons for the formation of codon usage patterns were inferred, in addition to the effect of mutation pressure, most of the driving forces of evolution may come from natural selection. 2–6 optimal codons were found in the chloroplast genome of 10 *Epimedium* species respectively. Meanwhile, *A. thaliana, P. trichocarpa,* and *S. cerevisiae* are relatively appropriate choices as receptors for the exogenous expression of chloroplast genes. This study provides a new perspective for understanding the codon usage patterns of chloroplast genomes in ten *Epimedium* species.

## Materials and methods

### Sequences acquisition and filtering

The complete chloroplast genomes of *E. koreanum* (MW_483096.1), *E. acuminatum* (MN_939630.1), *E. hunanense* (MW_483089.1), *E. sagittatum* (MT_560409.1), *E. leptorrhizum* (MT_560400.1), *E. pubescens* (MW_483097.1), *E. myrianthum* (MT_560401.1), *E. wushanense* (MN_857417.1), *E. brevicornu* (MN_803415.1), *E. coactum* (MT_560402.1) with genes annotations were downloaded from the National Center for Biotechnology Information (NCBI) database (https://www.ncbi.nlm.nih.gov). The number of original protein-coding sequences (CDS) of ten *Epimedium* species was 80, 84, 83, 83, 83, 85, 83, 83, 85 and 83 respectively (Table 1). To avoid analysis error, all CDS in chloroplast genomes of ten *Epimedium* species were extracted based on the following rules: (1) the length of the CDS should be greater than 300 bp [40]; (2) each CDS begins with a start codon (ATG), and ends with termination codons (TAG, TGA, TAA), (3) the number of the bases should be divided by three, (4) the CDS should not contain intermediate stop codon and wrong bases. After that, the GC content of three positions (GC1, GC2, GC3) were calculated by the CUSP program in EMBOSS explorer (http://emboss.toulouse.inra.fr./).

### Analysis of relative synonymous codon usage (RSCU) and relative synonymous codon usage frequency (RFSC)

The RSCU value refers to the ratio of the observed usage frequency of the codon to the expected usage frequency of all codons [41]. The RSCU values for all CDS of ten *Epimedium* species were calculated according to formula (1)

1 $$\textrm{RSC}U=\frac{x_{ij}}{\sum_j^{n_i}{x}_{ij}}{n}_i$$

where *x*_*ij*_ represents the frequency of codon *j* encoding for the *i* th amino acid, and *n*_*i*_ represents the number of synonymous codons encoding the *i* th amino acid. If the RSCU value of a codon equals 1.0 that indicates no codon usage bias and it is chosen equally with other synonymous codons. When the RSCU value is greater than 1.0, it is understood that the codon has a strong positive usage bias. In contrast, the RSCU value is lesser than 1.0, it is understood that the codon has a negative usage bias [42].

The RFSC value is the ratio of the actual observed number of a codon to the number of all synonymous codons. The RFSC values were calculated according to formula (2)

2 $$\textrm{RFSC}=\frac{x_{ij}}{\sum_j^{n_i}{x}_{ij}}$$

where *x*_*ij*_ represents the frequency of codon *j* encoding for the *i* th amino acid. If the RFSC of a codon is greater than 0.6 or more than 1.5 times the average frequency of synonymous codons, it can be defined as a high-frequency codon [43].

### Identification of putative optimal codons

ENc value is a significant parameter to evaluate the degree of codon usage bias. The ENc values range from 20 (only one synonymous codon is used to encode amino acids) to 61 (every synonymous codon is used equally). The smaller the ENc value of a codon, the stronger the codon usage bias. The ENc value of each *Epimedium* species was calculated by CodonW 1.4.2 software (http://codonw.sourceforge.net/). The chloroplast genes of each *Epimedium* species were reordered from low to high according to the ENc values. The top and bottom 5% of genes were selected as high and low expression datasets, and the RSCU values of each dataset were calculated by CodonW 1.4.2 respectively. Optimal codons were identified by ΔRSCU method. ΔRSCU of a codon is the difference between RSCU_high_ and RSCU_low_. If the ΔRSCU value is greater than or equal to 0.08 and the absolute of RSCU in a high or low expression dataset is greater than 1, it can be defined as an optimal codon [44].

### Comparative analysis of codon usage frequency

The codon usage frequency data of four model organisms were downloaded from Codon Usage Database. *Arabidopsis thaliana* (http://www.kazusa.or.jp/codon/cgi-bin/showcodon.cgi?species=3702), *Populus trichocarpa* (http://www.kazusa.or.jp/codon/cgi-bin/showcodon.cgi?species=3694), *Escherichia coli* (http://www.kazusa.or.jp/codon/cgi-bin/showcodon.cgi?species=199310), *Saccharomyces cerevisiae* (http://www.kazusa.or.jp/codon/cgibin/showcodon.cgi?species=4932). The codon usage frequency of ten *Epimedium* species was calculated by EMBOSS Explorer online program (https://www.bioinformatics.nl/emboss-explorer/). Moreover, the ratio of codon usage frequency for ten *Epimedium* species to four model species was computed. If the ratio is ≥2 or ≤ 0.5, the difference in codon usage is remarkable between the two organisms [45].

### ENc-GC3s plot analysis

GC3s is a noteworthy index of the nucleotide composition, which refers to the contents of guanine(G) and cytosine(C) at the third position of codons excluding Met and Trp. To explore the influencing factors of codon usage bias, the ENc-plot was drawn with GC3s as abscissa and ENc as ordinate. The expected ENc value was calculated by the formula (3) [46], and S represents GC3s. If codon usage bias is mostly affected by mutation pressure, the genes will be on or near the standard curve. On the contrary, if codon usage bias is influenced by natural selection, the genes will locate below the expected curve [47].

3 $$ENc=2+S+\frac{29}{S^2+{\left(1-S\right)}^2}$$

### PR2-plot analysis

The Parity Rule 2 plot analysis is usually used for estimating the influence of mutation pressure and natural selection on codon preference. It is a graphical analysis that reveals the composition of the bases at the third position of each codon. We established the graphic with A3/(A3 + T3) as the y-axis and G3/(G3 + C3) as the x-axis [48]. The points around the central point (A = T, G = C) illustrate the degree and direction of base deviation [49]. The center point means that there is no deviation between natural selection and mutation pressure. If the genes areis evenly distributed around the central point, it is considered that the codon bias may be entirely caused by mutation pressure.

### Neutrality plot analysis

Neutrality plot analysis is used to estimate the degree of influence between mutation pressure and natural selection on codon usage bias [50]. The scatter diagram was created with GC12 as ordinate and GC3 as abscissa. GC12 was the average GC content at the first and second positions of the codon. GC3 of each chloroplast gene of *Epimedium* species was calculated by Perl script (http://GitHub - hxiang1019/calc_GC_content). The coefficient of regression curve is close to or equal to 1, indicating that mutation pressure is the main factor of codon usage bias. Conversely, the coefficient near to or equal to 0 means that natural selection is the main factor of codon usage bias [51].

## Supplementary Information

**Additional file 1:** **Table S1.** RSCU and RFSC values of the codons in chloroplast genomes of ten Epimedium species.

**Additional file 2:** **Table S2.** The RSCU values and Δ RSCU value in the high and low expression library of the ten Epimedium species.

**Additional file 3:** **Table S3.** Comparison of codon usage frequency between ten Epimedium species and four model organisms.

## Data Availability

The chloroplast genome datasets generated and analyzed in this study are available in the NCBI, https://www.ncbi.nlm.nih.gov/nuccore/?term=Epimedium+Chloroplast+genome, and supplementary files.

## Abbreviations

A: Adenine
G: Guanine
C: Cytosine
U: Uracil
T: Thymine
A3,T3,G3,C3: The content of A,T, G, and C at the third condon position
GC1,GC2,GC3: The G + C content at the first, second, third condon positions
GC12: The average GC content at the first and second condon positions
RSCU: Relative synonymous codon usage
RFSC: Relative synonymous codon usage frequency
ENc: Effective number of codons
PR2: Parity Rule2
NCBI: National Center for Biotechnology Information.
